# Supplementary figures and images for: Physical activity, sedentary behavior and pancreatitis risk: Mendelian randomization study
Source: PLoS One. 2023 Jul 19;18(7):e0287810. doi: 10.1371/journal.pone.0287810 (PMC10355380; doi:10.1371/journal.pone.0287810)

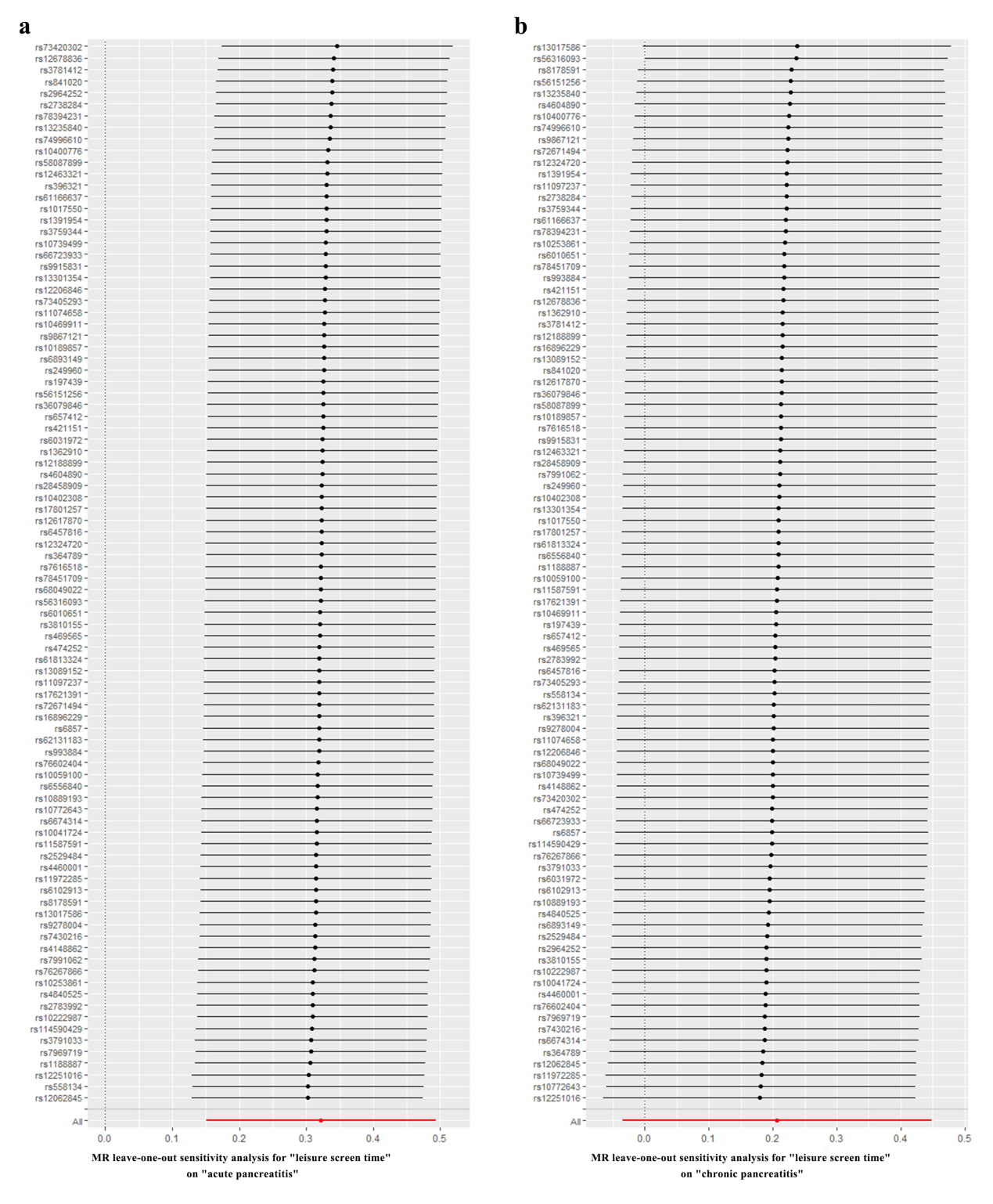


S1 Fig . Leave-one-out analysis for leisure screen time on acute/ chronic pancreatitis.

Supplement: S1 Fig — (DOCX) [file pone.0287810.s002.docx]
