# Supplementary material for: Physical activity, sedentary behavior and pancreatitis risk: Mendelian randomization study
Source: PLoS One. 2023 Jul 19;18(7):e0287810. doi: 10.1371/journal.pone.0287810 (PMC10355380; doi:10.1371/journal.pone.0287810)
Supplement: Attachment — Submitted filename: PONE-D-23-04358_reviewer_Comments.pdf [file pone.0287810.s003.pdf]

PLOS ONE

physical activity, sedentary behavior and pancreatitis risk: Mendelian randomization study

--Manuscript Draft--

**Manuscript Number:**	PONE-D-23-04358
**Article Type:**	Research Article
**Full Title:**	physical activity, sedentary behavior and pancreatitis risk: Mendelian randomization study
**Short Title:**	physical activity, sedentary behavior and pancreatitis risk: Mendelian randomization study
**Corresponding Author:**	Yanzhang Tian<br>Shanxi Bethune Hospital<br>Taiyuan, CHINA
**Keywords:**	physical activity; sedentary behavior; acute pancreatitis; chronic pancreatitis; Leisure screen time
**Abstract:**	Background<br><br>Although observational studies have shown that physical activity (PA) is a protective factor for acute pancreatitis, the causal associations between PA/ sedentary behavior and acute pancreatitis (AP) and chronic pancreatitis (CP) remain unclear.<br><br>Methods<br><br>We used Mendelian randomization as a strategy to assess the causalities between exposures and outcomes by simulating randomized experiments with genetic variation. The collected genetic variants data of PA were from UK Biobank(N = 377,324), the data on sedentary behavior(N > 526,000) could be found in the GWAS catalog, and the data on AP(5,509 cases, 301,383 controls) and CP (3,002 cases, 301,383 controls) were from FinnGen. There were three PA-related activity patterns (moderate to vigorous physical activity [MVPA], accelerometer-based PA with average acceleration, [AccAve] and accelerometer-based PA with accelerations >425 milli-gravities, [Acc425]) and three sedentary behavior-related lifestyle patterns (Leisure screen time [LST], Sedentary commuting, Sedentary behavior at work). We used inverse variance weighted (IVW), weighted median and MR-Egger for the analysis of Mendelian randomization, followed by sensitivity tests with the Cochran Q test, MR-Egger intercepts analysis and MR-PRESSO.<br><br>Results<br><br>A causal relationship was found between LST and acute pancreatitis based on IVW analysis (odds ratios [OR] = 1.38, corresponding 95% confidence intervals [CI] = 1.16-1.64, p = 0.0002) and Sensitivity analysis showed no pleiotropy and heterogeneity of the result.<br><br>Conclusions:<br><br>Results show that reducing LST contributes to the prevention of acute pancreatitis, thereby reducing the health burden associated with it.
**Order of Authors:**	Ruiqi Ling
	Jiabao Qi
	Juan Liang
	Shaojian Mo
	Xifeng Fu
	Yanzhang Tian

Powered by Editorial Manager® and ProduXion Manager® from Aries Systems Corporation

Additional Information:	
**Question**	**Response**
**Financial Disclosure**<br><br>Enter a financial disclosure statement that describes the sources of funding for the work included in this submission. Review the submission guidelines for detailed requirements. View published research articles from *PLOS ONE* for specific examples.<br><br>This statement is required for submission and **will appear in the published article** if the submission is accepted. Please make sure it is accurate.<br><br>**Unfunded studies**<br>Enter: *The author(s) received no specific funding for this work.*<br><br>**Funded studies**<br>Enter a statement with the following details:<br>• Initials of the authors who received each award<br>• Grant numbers awarded to each author<br>• The full name of each funder<br>• URL of each funder website<br>• Did the sponsors or funders play any role in the study design, data collection and analysis, decision to publish, or preparation of the manuscript?<br>• **NO** - Include this sentence at the end of your statement: *The funders had no role in study design, data collection and analysis, decision to publish, or preparation of the manuscript.*<br>• **YES** - Specify the role(s) played.<br><br>* typeset	The author(s) received no specific funding for this work.
**Competing Interests**<br><br>Use the instructions below to enter a competing interest statement for this submission. On behalf of all authors, disclose any competing interests that could be perceived to bias this work—acknowledging all financial support and any other relevant financial or non-financial competing interests.	The authors declare no conflict of interest.

Powered by Editorial Manager® and ProduXion Manager® from Aries Systems Corporation

This statement is required for submission and **will appear in the published article** if the submission is accepted. Please make sure it is accurate and that any funding sources listed in your Funding Information later in the submission form are also declared in your Financial Disclosure statement.<br><br>View published research articles from *PLOS ONE* for specific examples.<br><br>**NO authors have competing interests**<br><br>Enter: *The authors have declared that no competing interests exist.*<br><br>**Authors with competing interests**<br><br>Enter competing interest details beginning with this statement:<br><br>*I have read the journal's policy and the authors of this manuscript have the following competing interests: [insert competing interests here]*<br><br>* typeset	
**Ethics Statement**<br><br>Enter an ethics statement for this submission. This statement is required if the study involved:<br><br>• Human participants<br>• Human specimens or tissue<br>• Vertebrate animals or cephalopods<br>• Vertebrate embryos or tissues<br>• Field research<br><br>Write "N/A" if the submission does not require an ethics statement.<br><br>General guidance is provided below. Consult the submission guidelines for detailed instructions. **Make sure that all information entered here is included in the**	N/A

**Methods section of the manuscript.**<br><br>**Format for specific study types**<br><br>**Human Subject Research (involving human participants and/or tissue)**<br>• Give the name of the institutional review board or ethics committee that approved the study<br>• Include the approval number and/or a statement indicating approval of this research<br>• Indicate the form of consent obtained (written/oral) or the reason that consent was not obtained (e.g. the data were analyzed anonymously)<br><br>**Animal Research (involving vertebrate animals, embryos or tissues)**<br>• Provide the name of the Institutional Animal Care and Use Committee (IACUC) or other relevant ethics board that reviewed the study protocol, and indicate whether they approved this research or granted a formal waiver of ethical approval<br>• Include an approval number if one was obtained<br>• If the study involved *non-human primates*, add *additional details* about animal welfare and steps taken to ameliorate suffering<br>• If anesthesia, euthanasia, or any kind of animal sacrifice is part of the study, include briefly which substances and/or methods were applied<br><br>**Field Research**<br><br>Include the following details if this study involves the collection of plant, animal, or other materials from a natural setting:<br>• Field permit number<br>• Name of the institution or relevant body that granted permission	
**Data Availability**<br><br>Authors are required to make all data underlying the findings described fully available, without restriction, and from the time of publication. PLOS allows rare exceptions to address legal and ethical concerns. See the PLOS Data Policy and FAQ for detailed information.	Yes - all data are fully available without restriction

Powered by Editorial Manager® and ProduXion Manager® from Aries Systems Corporation

A Data Availability Statement describing where the data can be found is required at submission. Your answers to this question constitute the Data Availability Statement and **will be published in the article**, if accepted.<br><br>**Important:** Stating 'data available on request from the author' is not sufficient. If your data are only available upon request, select 'No' for the first question and explain your exceptional situation in the text box.<br><br>Do the authors confirm that all data underlying the findings described in their manuscript are fully available without restriction?	
**Describe where the data may be found in full sentences. If you are copying our sample text, replace any instances of XXX with the appropriate details.**<br><br>• If the data are **held or will be held in a public repository**, include URLs, accession numbers or DOIs. If this information will only be available after acceptance, indicate this by ticking the box below. For example: *All XXX files are available from the XXX database (accession number(s) XXX, XXX.*).<br>• If the data are all contained **within the manuscript and/or Supporting Information files**, enter the following: *All relevant data are within the manuscript and its Supporting Information files.*<br>• If neither of these applies but you are able to provide **details of access elsewhere**, with or without limitations, please do so. For example:<br><br>*Data cannot be shared publicly because of [XXX]. Data are available from the XXX Institutional Data Access / Ethics Committee (contact via XXX) for researchers who meet the criteria for access to confidential data.*<br><br>*The data underlying the results*	All the data comes from online databases,The patients/participants provided their written informed consent to participate in this study.

presented in the study are available from (include the name of the third party and contact information or URL).<br>• This text is appropriate if the data are owned by a third party and authors do not have permission to share the data.<br><br>* typeset	
Additional data availability information:	

Click here to access/download;Manuscript;manuscript.docx

physical activity, sedentary behavior and pancreatitis risk: Mendelian randomization study

Ruiqi Ling[1], Juan Liang[2], Shaojian Mo[1], Jiabao Qi[1], Xifeng Fu[1*], Yanzhang Tian[1*]

[1]General Surgery Department, Shanxi Bethune Hospital/General Surgery Department,Third Hospital of Shanxi Medical University, Taiyuan, China,

[2]The First Clinical Medical School, Shanxi Medical University, Taiyuan, China,

Correspondence: Yanzhang Tian, General Surgery Department, Shanxi Bethune Hospital/General Surgery Department,Third Hospital of Shanxi Medical University, Taiyuan, 030032, China, Email: tyz2030@163.com

Xifeng Fu, General Surgery Department, Shanxi Bethune Hospital/General Surgery Department,Third Hospital of Shanxi Medical University, Taiyuan, 030032, China, Email: fxfyisheng@163.com

Abstract

Background: Although observational studies have shown that physical activity (PA) is a protective factor for acute pancreatitis, the causal associations between PA/ sedentary behavior and acute pancreatitis (AP) and chronic pancreatitis (CP) remain unclear.

Methods: We used Mendelian randomization as a strategy to assess the causalities between exposures and outcomes by simulating randomized experiments with genetic variation. The collected genetic variants data of PA were from UK Biobank(N = 377,324), the data on sedentary behavior(N > 526,000) could be found in the GWAS catalog, and the data on AP(5,509 cases, 301,383 controls) and CP (3,002 cases, 301,383 controls) were from FinnGen. There were three PA-related activity patterns (moderate to vigorous physical activity [MVPA], accelerometer-based PA with average acceleration, [AccAve] and accelerometer-based PA with accelerations >425 milli-gravities, [Acc425]) and three sedentary behavior-related lifestyle patterns (Leisure screen time [LST], Sedentary commuting, Sedentary behavior at work). We used inverse variance weighted (IVW), weighted median and MR-Egger for the analysis of Mendelian randomization, followed by sensitivity tests with the Cochran Q test, MR-Egger intercepts analysis and MR-PRESSO.

Results: A causal relationship was found between LST and acute pancreatitis based on IVW analysis (odds ratios [OR] = 1.38, corresponding 95% confidence intervals [CI] = 1.16-1.64, p = 0.0002) and Sensitivity analysis showed no pleiotropy and heterogeneity of the result.

Conclusions: Results show that reducing LST contributes to the prevention of acute

pancreatitis, thereby reducing the health burden associated with it.

Keywords： physical activity; sedentary behavior; acute pancreatitis; chronic pancreatitis; Leisure screen time

Introduction

PA is a vital part of people's lives and is closely related to healthy [1]. Along with lifestyle and working changes, PA is gradually decreasing and other insalubrious lifestyles such as sedentary behavior are increasing. The rapid global movement of coronavirus disease 2019 (COVID-19) has further aggravated this problem [2]. New evidence has been shown PA helps prevent tumors [3], diabetes [4], cardiovascular disease [5], mental disorders [6] and other diseases[7]. Sedentary behavior is characterized by low energy expenditure activities (metabolic equivalents [METs] < 2.0), mostly in a sitting or supine position [8]. It has been confirmed to be a risk factor for obesity [9], autoimmune disease [10] and cancer [11], which has become a health burden and even influence mortality [12].

AP is the most common Gastrointestinal emergency in advanced countries [13]. The course of acute pancreatitis is difficult to predict despite various assessment criteria [14]. Although with the diversification of treatments, approximately 20% of patients develop moderate to severe cases and even die[13]. More than 3% of cases of acute pancreatitis progress to chronic pancreatitis within 3-8 years [15]. CP is a fibroinflammatory syndrome of the exocrine pancreas. With increasing pain, leading to diabetes and a higher risk of cancer, chronic pancreatitis greatly deteriorates life quality [15] [16]. In order to reduce the health burden of pancreatitis, early prevention is

essential. Previous studies have confirmed that smoking and alcohol consumption are risk factors for pancreatitis [17]. A recent observational study suggests that physical activity can reduce the risk for acute pancreatitis [18]. However, there is still a void regarding the causal relationship between PA/ sedentary behavior and pancreatitis. Mendelian randomization (MR) is an important strategy to demonstrate causality between exposure and outcome [19]. Instrumental variables used by MR to evaluate exposure-outcome relationships is genetic variants, Statistical methods for minimizing the impact of confounders [20]. At conception, genetic variants are randomly assigned and are not subject to disease progression, so reverse causality can be largely avoided [21]. The evidence level of randomized controlled trials (RCT) is highest in evidence-based medicine, but for ethical and economic reasons, RCT is generally feasible[22]. In the absence of RCT, MR has a higher level of evidence than observational studies. The purpose of this study was to reveal whether PA/sedentary behavior is causally related to pancreatitis based on the results of two samples of MR scanning.

Methods

Data sources

The AP genome-wide association study (GWAS) data which has more than 377,000 participants was from UK Biobank[23]. Three of phenotypes were selected, including moderate to MVPA, AccAve and Acc425. participants used an internationally standardized questionnaire to assess the physical activity, those moderate and high activity groups were classified into MVPA (N = 377,324) [23] [24]. Accelerometer-

based PA required participants to wear an Axivity AX3 accelerometer for at least 72h in a week [25]. There are two categories of data measured from accelerometer which were classified as AccAve (N = 91,084) and Acc425 (N = 90,667) [23].

GWAS data for sedentary behavior were referenced from a recent high-quality meta-analysis that included GWAS data from 51 studies in which we selected data on sedentary behavior of European ancestry[26]. The phenotypes were classified as LST (N = 526,725), Sedentary commuting (N = 159,606), Sedentary behavior at work (N = 372,609). Detailed definitions of the three phenotypes can be found from the supplementary data in Zhe Wang study[26]. The data can be found on GWAS catalog (https://www.ebi.ac.uk/gwas/).

The acute/chronic pancreatitis datasets we used are from FinnGen (https://r8.finngen.fi/), all data were updated in December 2022. The reason we used FinnGen datasets is to rule out the possibility of overlapping samples which may cause Sample overlap bias. There are 5,509 cases and 301,383 controls in the statistics of acute pancreatitis data. We also collected 3,002 cases and 301,383 controls for chronic pancreatitis, all participants own ancestry of European.

Study design

Exposure related single-nucleotide polymorphisms (SNPs) were used as IVs in MR study. To perform MR analysis, three assumptions must be met by IVs (Fig1): 1. There is a strong association between exposure and SNPs; 2. There should be no association between SNPs and potential confounders 3. SNPs have something to do with outcome only through exposure[19]. SNPs that were significantly ($P < 5 \times 10^{-8}$) associated with

PA/ sedentary behavior were selected, after we clumped these SNPs in linkage disequilibrium(LD, Clumping window: 10000kb, Clumping r^2 cutoff: 0.001) and used Pheno Scanner V2 (http://www.phenoscanner.medschl.cam.ac.uk/) to eliminate confounding SNPs (smoking and alcohol use related). For phenotypes with fewer than 3 SNPs, we rescreened with a relaxed P value of less than 5×10^{-7}, all other screening conditions unchanged[27]. The final SNPs of MVPA, AccAve, Acc425, LST, Sedentary commuting and Sedentary behavior at work are 16, 7, 4, 92, 3, 6 and all SNPs F-statistics >10 (The Supplementary table 1-6 provides details about SNPs).

Fig1. Instrumental variables fit the assumptions of MR. SNPs: single-nucleotide polymorphisms.

Mendelian randomization and Sensitivity analysis

The causal effects of PA/ sedentary behaviors on AP and CP were analyzed by IVW method, weighted median with MR-Egger was used as a supplement. If there were no heterogeneity and pleiotropy in IVs, the fixed-effects model analysis using IVW was the most accurate, but when heterogeneity was present, the random-effects model of IVW or methods of weighted median is preferred. In addition, It is suggested to use MR-Egger analysis instead of IVW and weighted median in the case of pleiotropy of SNPs [28]. Cochran Q test was performed to test for heterogeneity and the funnel plot is used as a supplement. horizontal pleiotropy of SNPs was tested by MR-Egger intercept analysis and MR pleiotropy residual sum and outlier (MR-PRESSO) was

conducted for assessing vertical pleiotropy and detection of biased SNPs ($p < 0.05$). MR-PRESSO was not feasible when there were less than or equal to three SNPs. For the presence of biased SNPs, MR and sensitivity analysis were repeated after excluding bias. At last, leave one out analysis was used to assess the robustness of the analysis results[29].

Statistical analysis

All statistical analyses were performed in R 4.2.2 using the R package TwoSampleMR (version 0.5.6). Because multiple independent analyses were performed, the P value ($p < 0.0083$: 0.05/6) after Bonferroni correction was used as the criterion for significance. The results of MR Analysis were shown in odds ratios (OR) with corresponding 95% confidence intervals (CI). Statistical power was calculated using mRnd(https://shiny.cnsgenomics.com/mRnd/)

Results

PA/ sedentary behavior and AP

Inverse variance weighted (IVW) Analysis showed that only LST had a causal relationship with acute pancreatitis (OR = 1.38, 95CI% = 1.16-1.64, p = 0.0002) and the statistical power was 0.99. Two other sedentary behavior-related phenotypes have been shown to reduce the risk of AP(Fig2). Other outcomes indicated that all three phenotypes of PA reduced the risk of acute pancreatitis (MVPA: OR = 0.45, 95CI% = 0.19-1.06, p = 0.0682; AccAve: OR = 0.96, 95CI% = 0.89-1.03, p = 0.2737; Acc425: 0.40, 95CI% = 0.14-1.18, p = 0.0980). The results of weighted median and MR-Egger

were listed in Supplementary Table S7.

Fig2. causal effects of PA/sedentary behavior on acute pancreatitis by IVW. PA: physical activity; MVPA: moderate to vigorous physical activity; AccAve: accelerometer-based PA (average acceleration); Acc425: accelerometer-based PA (fraction of time with accelerations >425 milli-gravities); LST: leisure screen time; IVW:inverse variance weighted.

PA/ sedentary behavior and CP

In total, neither the phenotypes of PA nor sedentary behavior were causally linked to CP. MVPA, AccAve and Acc425 play active roles for prevention of CP in IVW analysis (Fig3). LST and Sedentary commuting increased the risk of CP but Sedentary behavior at work reduced the risk. Weighted median and MR-Egge results also can be found in Supplementary Table 7.

Fig3. causal effects of PA/sedentary behavior on acute pancreatitis by IVW. PA: physical activity; MVPA: moderate to vigorous physical activity; AccAve: accelerometer-based PA (average acceleration); Acc425: accelerometer-based PA (fraction of time with accelerations >425 milli-gravities); LST: leisure screen time; IVW: inverse variance weighted.

Sensitivity analyses

To further evaluate the results' precision, sensitivity analyses, including MR-Egger intercept analysis, Cochran Q test, and MR-PRESSO global test, were conducted. MR-Egger intercept and MR-PRESSO analysis indicated that the pleiotropy of result of LST to AP/CP doesn't exist (Fig4) and we can draw robust conclusions after examining (supplementary Fig1). In other results, there was no horizontal pleiotropy since all MR-Egger intercepts were $p > 0.05$. The results of Cochran Q test indicated the heterogeneity of results when studying the relationship between AccAve and CP (Q = 15.15, p = 0.02) and MR-PRESSO shwon rs34517439 biased the results. After we eliminated rs34517439, we re-performed MR Analysis and sensitivity analysis of AccAve and CP. This time, Cochran Q test (Q = 6.66, p = 0.25) and MR-PRESSO global test (p = 0.28) shown that there is no heterogeneity in the results. There was no either heterogeneity nor pleiotropy in the other results (Table 1).

Table 1. Sensitivity analysis of the causal association between PA/sedentary behavior and the risk of AP/CP.

Exposure	Outcome	MR-Egger		Cochran Q test		MR-PRESSO
		Intercept	p	Q value	P	p
MVPA	AP	0.04	0.38	20.44	0.16	0.17
	CP	-0.03	0.54	19.39	0.20	0.20
AccAve	AP	-0.06	0.21	4.06	0.67	0.68
	CP	-0.06	0.44	6.66	0.25	0.28
Acc425	AP	-0.59	0.18	4.82	0.19	0.27
	CP	-0.32	0.50	1.00	0.80	0.77
LST	AP	-0.002	0.85	86.47	0.61	0.62
	CP	0.01	0.53	99.67	0.25	0.25
Sedentary commuting	AP	0.02	0.86	0.29	0.86	-
	CP	-0.02	0.91	0.47	0.79	-
Sedentary behavior at work	AP	-0.03	0.46	3.62	0.61	0.63
	CP	-0.04	0.43	3.50	0.62	0.67

MVPA: moderate to vigorous physical activity; AccAve: accelerometer-based PA (average acceleration); Acc425: accelerometer-based PA (fraction of time with

accelerations >425 milli-gravities); LST: leisure screen time; AP: acute pancreatitis; CP: chronic pancreatitis.

Fig4. Sensitivity analyses of MR results about LST to acute/chronic pancreatitis: a, b: Scatter plots and funnel plots show genetically predicted of LST on acute pancreatitis; c, d: genetically predicted LST on chronic pancreatitis. LST: Leisure screen time.

Discussion

To date, no studies have investigated the causal relationship between PA/ sedentary behavior and AP/CP.

PA and sedentary behavior are the two most common lifestyle habits of people. As people pay more attention to health, research on PA and sedentary behavior has exploded in recent years. Although latest studies have identified that PA/ sedentary behavior is associated with cancer[30], various chronic diseases, and even some studies have shown that sedentary behavior can increase the severity of COVID-19 and the probability of hospitalization. However, studies on their association with gastrointestinal diseases are still scarce.

Pancreatitis is one of the most common gastrointestinal diseases. There was a reduction in the risk of AP among PA participants in a large observational study in China. Observational studies, however, are susceptible to confounding factors and reverse causality, and Asian findings may not generalize to European populations. There are even fewer studies on PA/ sedentary behavior and CP, and these research directions have focused on influence of prognosis. It is still difficult for patients with acute pancreatitis and chronic pancreatitis to have a good quality of life during their illness,

despite major improvements in treatment. Therefore, the prevention of pancreatitis is important and understanding the protective and risk factors of pancreatitis is urgent.

It was our study that first demonstrated causally that LST increases AP risk. It may caused by following reasons. firstly, LST people often have abnormal eating habits, they usually don’t have a habit of breakfast and the intake of vitamin and dietary fiber in the diet is low, fat composition is relatively high[31].These irregular eating habits can induce the formation of gallstones[32] and gallstones is the most common trigger of AP in advanced countries[33]. Secondly, A recent Mendelian randomization study has demonstrated that LST increases the risk of type 2 diabetes causally[34]. Dipeptidylpeptidase-4 (DPP4) inhibitors and Glucagon-like peptide-1 receptor agonist (GLP-1RAs) are important treatments for T2D.These medicines may increase the risk of pancreatitis[35, 36]. Last but not least, sedentary behavior will increase risk of autoimmune disease[10] which also a risk factor for AP in European populations[13].

In our study, it was indeed not shown that PA would affect AP/CP. This contradicts the findings of previous observational study[18]. We speculate that this may caused by people who exercise regularly will have less bad lifestyle habits such as smoking and drinking. Smoking and drinking are risk factors for pancreatitis which cause the bias[16]. Qilin Qian's study confirmed that physical activity reduces the risk of gallstone disease[37],which may cause bias of observational study.

We used SNPs instead of exposure to investigate the causal relationship between exposures and outcomes. Compared with observational studies, this strategy can effectively avoid interference of confounders and reverse causality. In the selection of

GWAS data, we selected data with European ancestry, which can largely avoid the bias caused by different races. Different databases were used for the selection of GWAS data for exposures and outcomes to avoid bias due to overlapping samples. Our results have clinical implications, and reducing LST can effectively reduce the risk of AP, thereby reducing the health burden of European countries.

However, our study still has some limitations. In the first place, because all GWAS data were derived from participants of European ancestry, our results only apply to European populations and cannot be generalized to all humans. Moreover, detailed disease information was not available for participants in the course of the GWAS data collection, stratified analyses could not be performed and it is not possible to infer whether PA/ sedentary behavior has a causal link to disease severity in pancreatitis either. In addition, except for the MR estimates of LST and AP, the statistical power of the other MR Analysis results was low. which may lead to false negative results.

All in all, there are no causal relationship between PA and pancreatitis. It's LST rather than sedentary behavior increase the risk of AP causally but it has no causal connection with CP.

Declarations

Ethics approval and consent to participate

All the data comes from online databases,The patients/participants provided their written informed consent to participate in this study.

Consent for publication

Not applicable.

Availability of data and materials

The datasets presented in this study can be found in online，The names and links of the repositories can be found in the paper.

Competing interests

The authors declare no conflict of interest.

Funding

This study was supported by Shanxi Province 136 revitalization medical project (department of general surgery), Shanxi Provincial Department of Human Resources and Social Security (no. 20210002) and Shanxi Provincial Department of Science and Technology (no. 202104041101024)

Authors' contributions

YT, XF and RL conceived and designed the study protocol. RL, JQ and SM conducted the experiments and performed the statistical analysis. RL and JL wrote the paper. All authors have read and approved the final manuscript.

Acknowledgements

We want to acknowledge the participants and investigators of the UK Biobank and the FinnGen study.

1. Harridge SD, Lazarus NR. Physical Activity, Aging, and Physiological Function. Physiology (Bethesda). 2017;32(2):152-61. Epub 2017/02/24. doi: 10.1152/physiol.00029.2016. PubMed PMID: 28228482.

2. Hall G, Laddu DR, Phillips SA, Lavie CJ, Arena R. A tale of two pandemics: How will COVID-19 and global trends in physical inactivity and sedentary behavior affect one another? Prog Cardiovasc Dis. 2021;64:108-10. Epub 2020/04/12. doi: 10.1016/j.pcad.2020.04.005. PubMed PMID: 32277997; PubMed Central PMCID: PMCPMC7194897.

3. Wang Q, Zhou W. Roles and molecular mechanisms of physical exercise in cancer prevention and treatment. J Sport Health Sci. 2021;10(2):201-10. Epub 2020/08/02. doi: 10.1016/j.jshs.2020.07.008. PubMed PMID: 32738520; PubMed Central PMCID: PMCPMC7987556.

4. Magkos F, Hjorth MF, Astrup A. Diet and exercise in the prevention and treatment of type 2 diabetes mellitus. Nat Rev Endocrinol. 2020;16(10):545-55. Epub 2020/07/22. doi: 10.1038/s41574-020-0381-5. PubMed PMID: 32690918.

5. Force USPST, Mangione CM, Barry MJ, Nicholson WK, Cabana M, Coker TR, et al. Behavioral Counseling Interventions to Promote a Healthy Diet and Physical Activity for Cardiovascular Disease Prevention in Adults Without Cardiovascular Disease Risk Factors: US Preventive Services Task Force Recommendation Statement. JAMA. 2022;328(4):367-74. Epub 2022/07/27. doi: 10.1001/jama.2022.10951. PubMed PMID: 35881115.

6. Iso-Markku P, Kujala UM, Knittle K, Polet J, Vuoksimaa E, Waller K. Physical activity as a

protective factor for dementia and Alzheimer's disease: systematic review, meta-analysis and quality assessment of cohort and case-control studies. Br J Sports Med. 2022;56(12):701-9. Epub 2022/03/19. doi: 10.1136/bjsports-2021-104981. PubMed PMID: 35301183; PubMed Central PMCID: PMCPMC9163715.

7. Kandola A, Ashdown-Franks G, Hendrikse J, Sabiston CM, Stubbs B. Physical activity and depression: Towards understanding the antidepressant mechanisms of physical activity. Neurosci Biobehav Rev. 2019;107:525-39. Epub 2019/10/06. doi: 10.1016/j.neubiorev.2019.09.040. PubMed PMID: 31586447.

8. Tremblay MS, Aubert S, Barnes JD, Saunders TJ, Carson V, Latimer-Cheung AE, et al. Sedentary Behavior Research Network (SBRN) - Terminology Consensus Project process and outcome. Int J Behav Nutr Phys Act. 2017;14(1):75. Epub 2017/06/11. doi: 10.1186/s12966-017-0525-8. PubMed PMID: 28599680; PubMed Central PMCID: PMCPMC5466781.

9. Bluher M. Obesity: global epidemiology and pathogenesis. Nat Rev Endocrinol. 2019;15(5):288-98. Epub 2019/03/01. doi: 10.1038/s41574-019-0176-8. PubMed PMID: 30814686.

10. Pinto AJ, Roschel H, de Sa Pinto AL, Lima FR, Pereira RMR, Silva CA, et al. Physical inactivity and sedentary behavior: Overlooked risk factors in autoimmune rheumatic diseases? Autoimmun Rev. 2017;16(7):667-74. Epub 2017/05/10. doi: 10.1016/j.autrev.2017.05.001. PubMed PMID: 28479487.

11. Kerr J, Anderson C, Lippman SM. Physical activity, sedentary behaviour, diet, and cancer: an update and emerging new evidence. Lancet Oncol. 2017;18(8):e457-e71. Epub 2017/08/02. doi: 10.1016/S1470-2045(17)30411-4. PubMed PMID: 28759385.

12. Ekelund U, Tarp J, Steene-Johannessen J, Hansen BH, Jefferis B, Fagerland MW, et al. Dose-response associations between accelerometry measured physical activity and sedentary time and all cause mortality: systematic review and harmonised meta-analysis. BMJ. 2019;366:l4570. Epub 2019/08/23. doi: 10.1136/bmj.l4570. PubMed PMID: 31434697; PubMed Central PMCID: PMCPMC6699591 and declare: no support from any organisation for the submitted work; no financial relationships with any organisations that might have an interest in the submitted work in the previous three years; no other relationships or activities that could appear to have influenced the submitted work.

13. Boxhoorn L, Voermans RP, Bouwense SA, Bruno MJ, Verdonk RC, Boermeester MA, et al. Acute pancreatitis. Lancet. 2020;396(10252):726-34. Epub 2020/09/07. doi: 10.1016/S0140-6736(20)31310-6. PubMed PMID: 32891214.

14. Di MY, Liu H, Yang ZY, Bonis PA, Tang JL, Lau J. Prediction Models of Mortality in Acute Pancreatitis in Adults: A Systematic Review. Ann Intern Med. 2016;165(7):482-90. Epub 2016/07/28. doi: 10.7326/M16-0650. PubMed PMID: 27454310.

15. Beyer G, Habtezion A, Werner J, Lerch MM, Mayerle J. Chronic pancreatitis. Lancet. 2020;396(10249):499-512. Epub 2020/08/18. doi: 10.1016/S0140-6736(20)31318-0. PubMed PMID: 32798493.

16. Vege SS, Chari ST. Chronic Pancreatitis. N Engl J Med. 2022;386(9):869-78. Epub 2022/03/03. doi: 10.1056/NEJMcp1809396. PubMed PMID: 35235728.

17. Petrov MS, Yadav D. Global epidemiology and holistic prevention of pancreatitis. Nat Rev Gastroenterol Hepatol. 2019;16(3):175-84. Epub 2018/11/30. doi: 10.1038/s41575-018-0087-5. PubMed PMID: 30482911; PubMed Central PMCID: PMCPMC6597260.

18. Pang Y, Kartsonaki C, Turnbull I, Guo Y, Yang L, Bian Z, et al. Metabolic and lifestyle risk factors for acute pancreatitis in Chinese adults: A prospective cohort study of 0.5 million people. PLoS Med. 2018;15(8):e1002618. Epub 2018/08/02. doi: 10.1371/journal.pmed.1002618. PubMed PMID: 30067849; PubMed Central PMCID: PMCPMC6070164.

19. Gala H, Tomlinson I. The use of Mendelian randomisation to identify causal cancer risk factors: promise and limitations. J Pathol. 2020;250(5):541-54. Epub 2020/03/11. doi: 10.1002/path.5421. PubMed PMID: 32154591.

20. Burgess S, Thompson SG. Multivariable Mendelian randomization: the use of pleiotropic genetic variants to estimate causal effects. Am J Epidemiol. 2015;181(4):251-60. Epub 2015/01/30. doi: 10.1093/aje/kwu283. PubMed PMID: 25632051; PubMed Central PMCID: PMCPMC4325677.

21. Davey Smith G, Hemani G. Mendelian randomization: genetic anchors for causal inference in epidemiological studies. Hum Mol Genet. 2014;23(R1):R89-98. Epub 2014/07/30. doi: 10.1093/hmg/ddu328. PubMed PMID: 25064373; PubMed Central PMCID: PMCPMC4170722.

22. Cornish AJ, Tomlinson IPM, Houlston RS. Mendelian randomisation: A powerful and inexpensive method for identifying and excluding non-genetic risk factors for colorectal cancer. Mol Aspects Med. 2019;69:41-7. Epub 2019/02/03. doi: 10.1016/j.mam.2019.01.002. PubMed PMID: 30710596; PubMed Central PMCID: PMCPMC6856712.

23. Klimentidis YC, Raichlen DA, Bea J, Garcia DO, Wineinger NE, Mandarino LJ, et al. Genome-wide association study of habitual physical activity in over 377,000 UK Biobank participants identifies multiple variants including CADM2 and APOE. Int J Obes (Lond). 2018;42(6):1161-76. Epub 2018/06/15. doi: 10.1038/s41366-018-0120-3. PubMed PMID:

29899525; PubMed Central PMCID: PMCPMC6195860.

24. Craig CL, Marshall AL, Sjostrom M, Bauman AE, Booth ML, Ainsworth BE, et al. International physical activity questionnaire: 12-country reliability and validity. Med Sci Sports Exerc. 2003;35(8):1381-95. Epub 2003/08/06. doi: 10.1249/01.MSS.0000078924.61453.FB. PubMed PMID: 12900694.

25. Doherty A, Jackson D, Hammerla N, Plotz T, Olivier P, Granat MH, et al. Large Scale Population Assessment of Physical Activity Using Wrist Worn Accelerometers: The UK Biobank Study. PLoS One. 2017;12(2):e0169649. Epub 2017/02/02. doi: 10.1371/journal.pone.0169649. PubMed PMID: 28146576; PubMed Central PMCID: PMCPMC5287488 study. PO has previously been a director of Axivity Ltd. NH has previously consulted for Axivity Ltd. The partners of DJ and PO own shares in Axivity. This does not alter our adherence to PLOS ONE policies on sharing data and materials.

26. Wang Z, Emmerich A, Pillon NJ, Moore T, Hemerich D, Cornelis MC, et al. Genome-wide association analyses of physical activity and sedentary behavior provide insights into underlying mechanisms and roles in disease prevention. Nat Genet. 2022;54(9):1332-44. Epub 2022/09/08. doi: 10.1038/s41588-022-01165-1. PubMed PMID: 36071172; PubMed Central PMCID: PMCPMC9470530 Lorentzon has received lecture or consulting fees from Amgen, Lilly, UCB Pharma, Radius Health, Meda, GE-Lunar and Santax Medico/Hologic. P.V. received an unrestricted grant from GlaxoSmithKline to build the CoLaus study. These authors played a role in individual studies that contributed to the meta-analysis, but not to the meta-analysis of GWAS studies, downstream experiments and analyses, or interpretation of the data. Hence, it is highly unlikely to have influenced the results of this study. The remaining authors declare no

competing interests.

27. Carrasquilla GD, Garcia-Urena M, Fall T, Sorensen TIA, Kilpelainen TO. Mendelian randomization suggests a bidirectional, causal relationship between physical inactivity and adiposity. Elife. 2022;11. Epub 2022/03/08. doi: 10.7554/eLife.70386. PubMed PMID: 35254260; PubMed Central PMCID: PMCPMC8975550.

28. Burgess S, Thompson SG. Interpreting findings from Mendelian randomization using the MR-Egger method. Eur J Epidemiol. 2017;32(5):377-89. Epub 2017/05/21. doi: 10.1007/s10654-017-0255-x. PubMed PMID: 28527048; PubMed Central PMCID: PMCPMC5506233.

29. Wu F, Huang Y, Hu J, Shao Z. Mendelian randomization study of inflammatory bowel disease and bone mineral density. BMC Med. 2020;18(1):312. Epub 2020/11/11. doi: 10.1186/s12916-020-01778-5. PubMed PMID: 33167994; PubMed Central PMCID: PMCPMC7654011.

30. Dixon-Suen SC, Lewis SJ, Martin RM, English DR, Boyle T, Giles GG, et al. Physical activity, sedentary time and breast cancer risk: a Mendelian randomisation study. Br J Sports Med. 2022;56(20):1157-70. Epub 2022/11/04. doi: 10.1136/bjsports-2021-105132. PubMed PMID: 36328784.

31. Byun D, Kim R, Oh H. Leisure-time and study-time Internet use and dietary risk factors in Korean adolescents. Am J Clin Nutr. 2021;114(5):1791-801. Epub 2021/07/15. doi: 10.1093/ajcn/nqab229. PubMed PMID: 34258617.

32. Di Ciaula A, Garruti G, Fruhbeck G, De Angelis M, de Bari O, Wang DQ, et al. The Role of Diet in the Pathogenesis of Cholesterol Gallstones. Curr Med Chem. 2019;26(19):3620-38.

Epub 2017/05/31. doi: 10.2174/0929867324666170530080636. PubMed PMID: 28554328; PubMed Central PMCID: PMCPMC8118138.

33. Roberts SE, Morrison-Rees S, John A, Williams JG, Brown TH, Samuel DG. The incidence and aetiology of acute pancreatitis across Europe. Pancreatology. 2017;17(2):155-65. Epub 2017/02/06. doi: 10.1016/j.pan.2017.01.005. PubMed PMID: 28159463.

34. Deng MG, Cui HT, Lan YB, Nie JQ, Liang YH, Chai C. Physical activity, sedentary behavior, and the risk of type 2 diabetes: A two-sample Mendelian Randomization analysis in the European population. Front Endocrinol (Lausanne). 2022;13:964132. Epub 2022/11/22. doi: 10.3389/fendo.2022.964132. PubMed PMID: 36407298; PubMed Central PMCID: PMCPMC9670309.

35. Smits MM, Van Raalte DH. Safety of Semaglutide. Front Endocrinol (Lausanne). 2021;12:645563. Epub 2021/07/27. doi: 10.3389/fendo.2021.645563. PubMed PMID: 34305810; PubMed Central PMCID: PMCPMC8294388.

36. Home P. Cardiovascular outcome trials of glucose-lowering medications: an update. Diabetologia. 2019;62(3):357-69. Epub 2019/01/05. doi: 10.1007/s00125-018-4801-1. PubMed PMID: 30607467.

37. Qian Q, Jiang H, Cai B, Chen D, Jiang M. Physical activity and risk of gallstone disease: A Mendelian randomization study. Front Genet. 2022;13:943353. Epub 2022/12/24. doi: 10.3389/fgene.2022.943353. PubMed PMID: 36561321; PubMed Central PMCID: PMCPMC9763559.

 Click here to access/download;Figure;figure1.tif

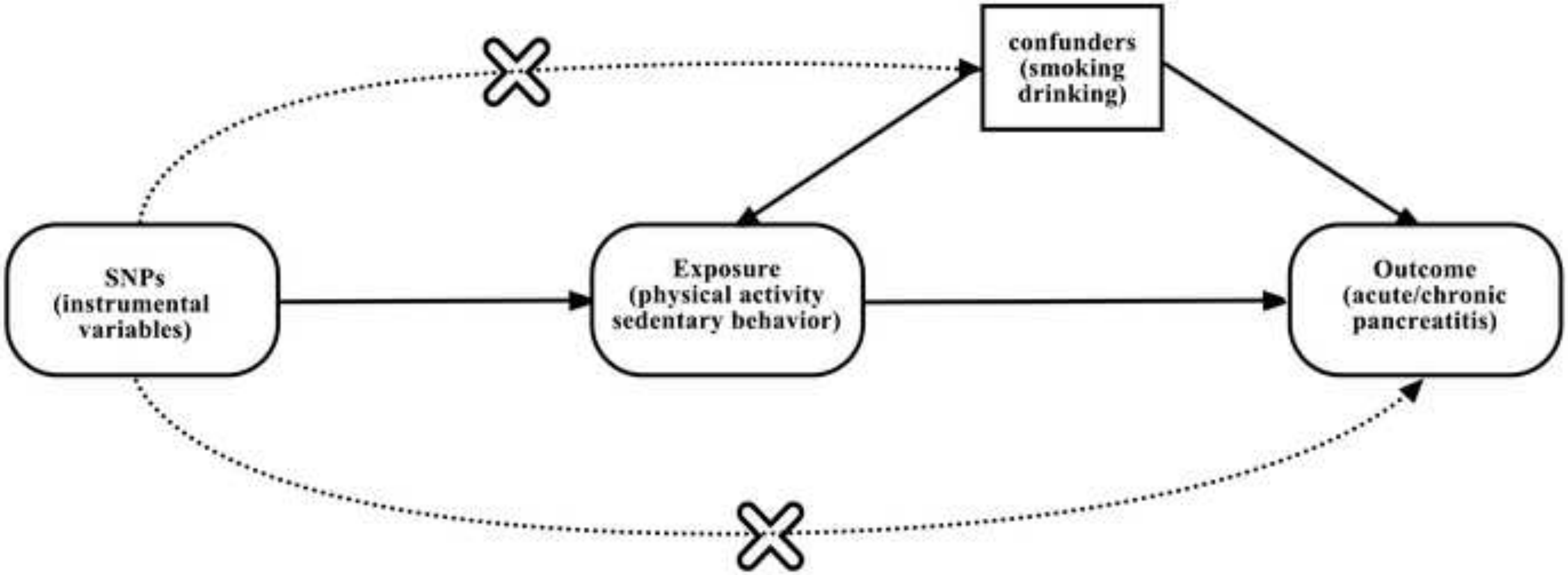

Click here to access/download;Figure;figure2.tif

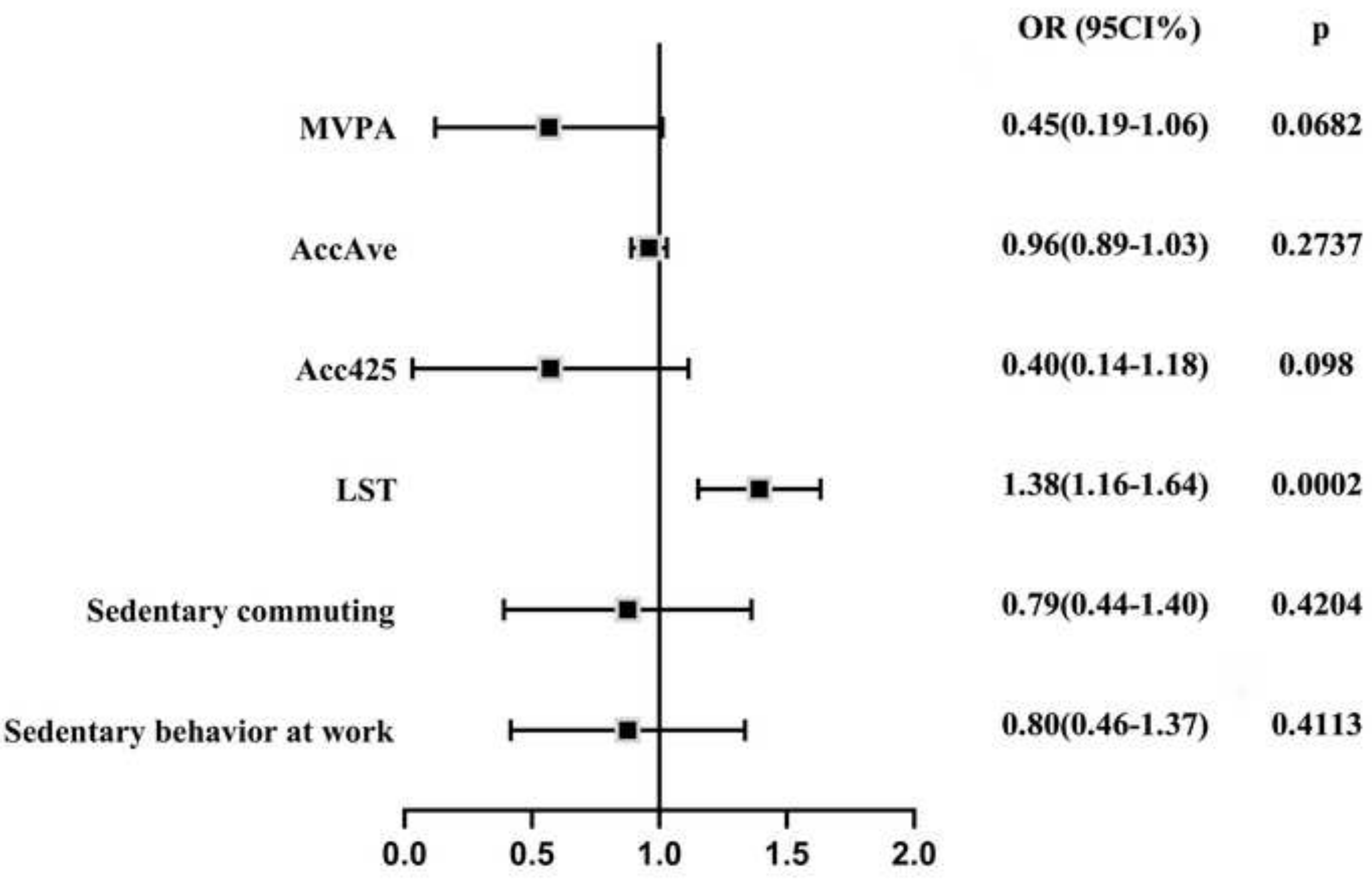

Figure
Click here to access/download;Figure;figure3.tif

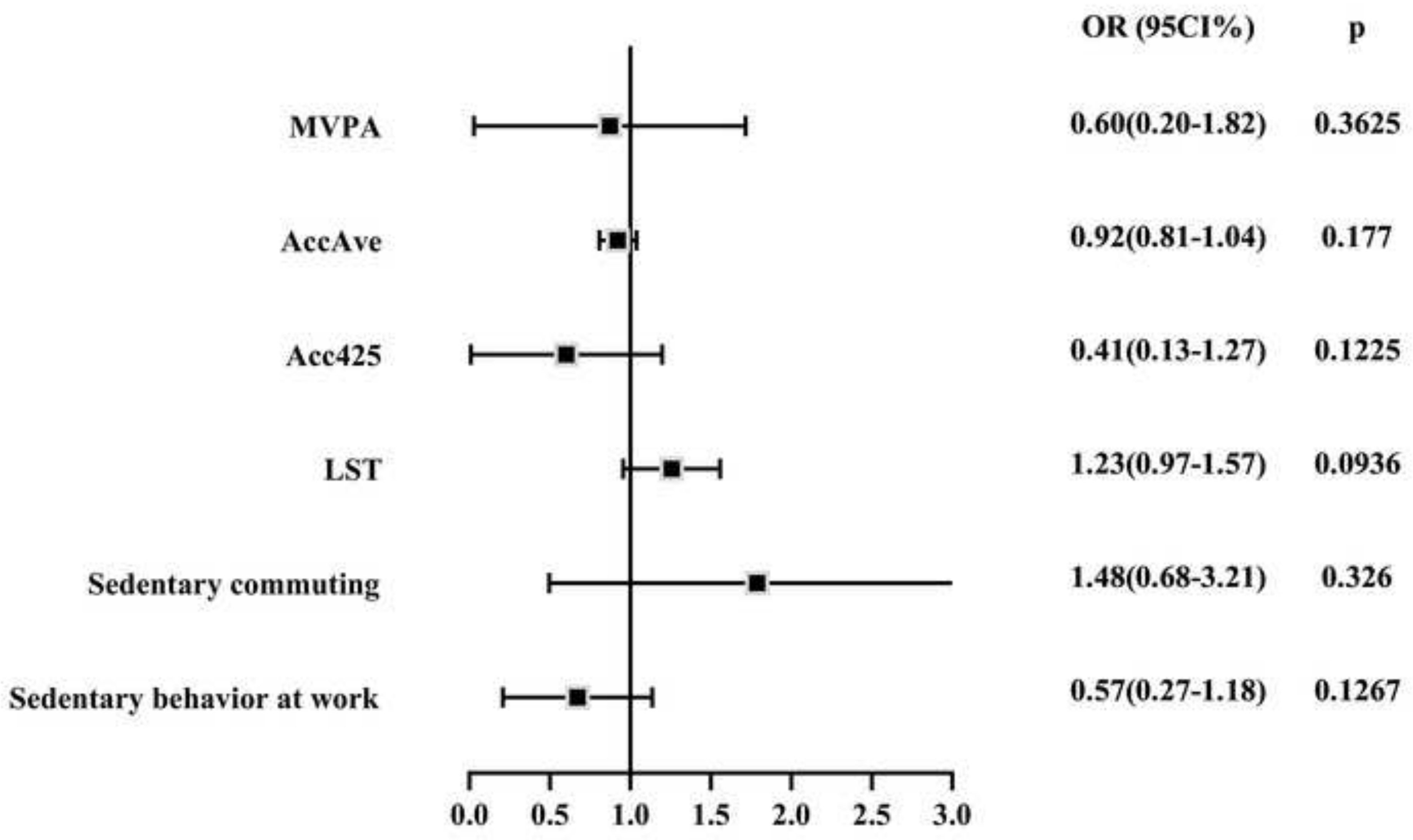

Figure

Click here to access/download;Figure;figure4.tif

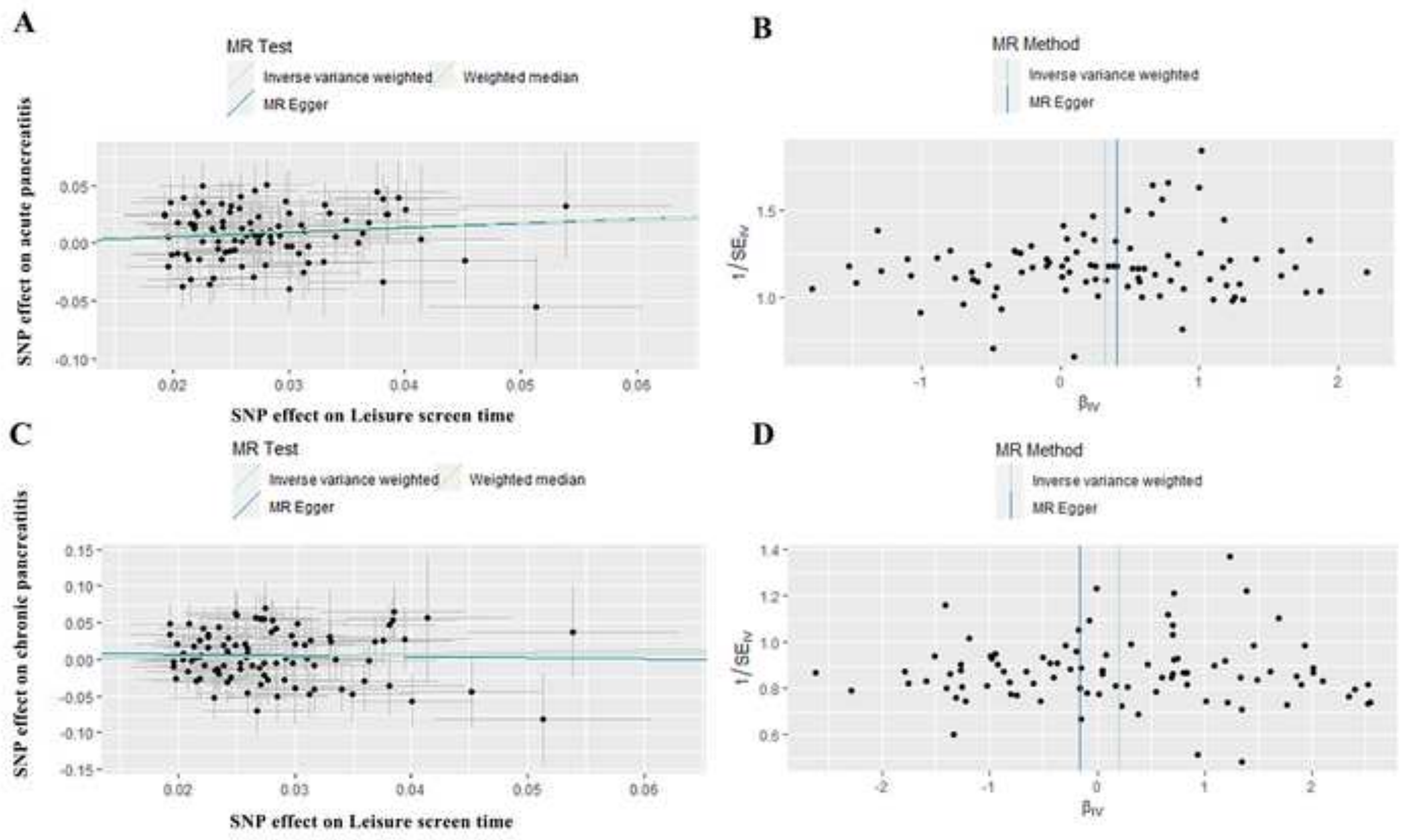

Cover Letter

Click here to access/download
Supporting Information
supplementary figure.docx

Click here to access/download
Supporting Information
supplementary tables.xls
