# Supplementary material for: Physical activity, sedentary behavior and pancreatitis risk: Mendelian randomization study
Source: PLoS One. 2023 Jul 19;18(7):e0287810. doi: 10.1371/journal.pone.0287810 (PMC10355380; doi:10.1371/journal.pone.0287810)
Supplement: Attachment — Submitted filename: Response to Reviewers.docx [file pone.0287810.s004.docx]

Dear academic editor and reviewers,

Thank you very much for your comments and professional advice These opinions help to improve the academic rigor of our article Based on your suggestion and request we have made corrected modifications to the revised manuscript. We hope that our work can be improved again Furthermore, we would like to show the details as follows:

**Academic editor:**

1. Please ensure that your manuscript meets PLOS ONE's style requirements, including those for file naming. The PLOS ONE style templates can be found at

https://journals.plos.org/plosone/s/file?id=wjVg/PLOSOne_formatting_sample_main_body.pdf and

https://journals.plos.org/plosone/s/file?id=ba62/PLOSOne_formatting_sample_title_authors_affiliations.pdf

2. We note that the grant information you provided in the ‘Funding Information’ and ‘Financial Disclosure’ sections do not match.

When you resubmit, please ensure that you provide the correct grant numbers for the awards you received for your study in the ‘Funding Information’ section.

3. Your ethics statement should only appear in the Methods section of your manuscript. If your ethics statement is written in any section besides the Methods, please move it to the Methods section and delete it from any other section. Please ensure that your ethics statement is included in your manuscript, as the ethics statement entered into the online submission form will not be published alongside your manuscript.

4. Please include captions for your Supporting Information files at the end of your manuscript, and update any in-text citations to match accordingly. Please see our Supporting Information guidelines for more information: http://journals.plos.org/plosone/s/supporting-information.

5. Please review your reference list to ensure that it is complete and correct. If you have cited papers that have been retracted, please include the rationale for doing so in the manuscript text, or remove these references and replace them with relevant current references. Any changes to the reference list should be mentioned in the rebuttal letter that accompanies your revised manuscript. If you need to cite a retracted article, indicate the article’s retracted status in the References list and also include a citation and full reference for the retraction notice.

**The author's answer:**

1. We have followed the magazine's requirements for the author information and title format in the manuscript and deleted other unnecessary sections to make sure that our manuscript meets PLOS ONE's style requirements.
2. We kindly request to modify our financial disclosure statement. Our research received funding from the Shanxi Provincial Department of Human Resources and Social Security (grant no. 20210002) and the Shanxi Provincial Department of Science and Technology (grant no. 202104041101024). The funding agency is represented by the abbreviation YT. Furthermore, we would like to clarify that the funders had no involvement in the study design, data collection, and analysis.
3. We have moved the ethics statement to the Methods section and deleted it from other section
4. We have added Supporting Information files at the end of oour manuscript and updated any in-text citations to match accordingly
5. We added a reference, which is the 19th reference in the full text, to add the rationale for our study. The reference list is complete and correct. We have not cited papers that have been retracted

Reviewer #1:

1- how to calculate sample size in the study

2- whats number of instruments is used for MVPA as small number may affect on the power of study

3- please write about Pleitropic effects of smoking and alcohol consumption and data sources as paragraph

4- whats about the effect of horizanatal pleiotropic on bias

5- you use three indicators for sedentary behaviour and three indicators for physical activity

6- at conclusion please write about the linking between physcial activity and AP and CP is determined or not

7- Are individuals of the UK Biobank were included in both the exposure and outcome datasets as this potential limitation which might introduce some bias in the causal estimates in the direction of the estimates of observational studies

8- you should give titles regarding the data sources as following :

*instrumentalvariable selection data

*data sources for acute and chronic pancreatitis

*data for smoking and drinking alcohol

9- why no figure shown illustating the study design and Study design to explain easily for the readers

10- why use abbreviation for physical activity i think not good this abbreviations as not specific

**The author's answer:**

1: We use data from UK Biobank and FinnGen as our sources. The sample size in the database remains constant, and in order to achieve greater statistical power, we have included all eligible data that meets the criteria. The calculation of sample size and statistical power is performed using the website [https://shiny.cnsgenomics.com/mRnd/. (page5](https://shiny.cnsgenomics.com/mRnd/.(page5) section of Instrumental variable selection data)

2: The number of instrumental variables (IVs) we have selected for MVPA is 16, which were carefully screened through strict selection steps. This quantity is not small and is similar to the number of MVPA experiments used as instruments in previous Mendelian randomization studies. However, as our outcome differs from those studies, there may be slight variations in the number of instrumental variables used. The statistical power we calculated of MVPA to acute/chronic pancreatitis were 0.32 and 0.12(page6 line 126-128)

3: Smoking and alcohol use are confounding factors, and if the selected instrumental variables are associated with them, the study would not meet the second assumption of Mendelian randomization (MR). Therefore, it is important to exclude SNPs that are associated with smoking and alcohol use to avoid confounding factors affecting the causal relationship. They only play confounding roles, so the data on smoking and alcohol use is unnecessary.(page 3 line 63-65)

4. we have added the effect of horizontal pleiotropic on bias(page7 line 142-147)

5: Regarding the selection of three phenotypes related to physical activity and sedentary behavior, we referred to all relevant GWAS and Mendelian randomization studies. We chose the three phenotypes that were most frequently used and representative in these studies as indicators for our analysis(page 5 line89-92)

6: physical activity has no causal relationship with acute/chronic pancreatitis, which we have added in the paper.(page8 line169-171)

7: the exposure date came from UK biobank and the outcome date was from FinnGen, They came from different countries so there was no bias due to sample overlap.(page2 line24-27)

8: we have reversed titles regarding the data sources, but the data didn't include data for smoking and drinking alcohol. Smoking and drinking alcohol only play confounding roles. And we ticked out the SNPs associated with them to make sure our MR won't be interfered by confounders.(page4-5 section of data sources)

9: We have added a figure(Fig 1) illustrating the study design and Study design to explain easily to the readers(page6 line131)

10: this abbreviation was not specific, so we have canceled the abbreviate physical to PA.

**Reviewer #2:**

This study is interesting. However, there are still some parts to be improved such as the figure 4 presentation (blurred), punctuation, and abstract writing.

1. Please enhance the result section in abstract with other important findings.

2. line 68-69 In which working system in the body the PA could reduce the risk for AP? Please explore this explanation in this paragraph concisely.

3. line 73 Do the genetic variants are chosen based on the genetic that involve in the pancreatitis development?

4. line 87 Mention the name of the questionnaire used in this study

5. line 221-222 Why author did not use the dietary intake data of each subject to be one of their co-variables?

6. line 251 Delete the dot (.)

7. line 252-254 Please add one sentence to inform the public recommendation based on the study findings.

**The author's answer:**

We have adjusted our picture4 to make it won’t blur(We added an additional figure, so Figure 4 is now referred to as Figure 5.)

1: We have enhanced the result section in the abstract by adding the relationship between physical activity/sedentary behavior and chronic pancreatitis.(page2 line 35-39)

2: Physical activity may modulate the endocrine effects of the pancreas, thereby reducing the risk of pancreatitis.(page 4 line 68-69)

3: Genetic variations also influence the development of pancreatitis.(page 4 line 75-76)

4: The questionnaire referred to is the international physical activity questionnaire (August 2002 version)(page5 89-92)

5: There are 2 reasons to explain why we didn't use the dietary intake data of each subject to be one of their co-variables (page12 line 240-242)

1. the data we collected do not have the dietary intake data. The database builders did not consider the collection of this part of the data.

2. The SNPs selected as the instrument variable do not have connections with dietary intake.

6: We have deleted the dot (.)(page13 line 271-273)

7: We have added one sentence to inform the public recommendation based on the study findings.(page13 line 275-276)
